# Cross-phenotype association mapping of the MHC identifies genetic variants that differentiate psoriatic arthritis from psoriasis

**DOI:** 10.1136/annrheumdis-2017-211414

**Published:** 2017-08-18

**Authors:** John Bowes, James Ashcroft, Nick Dand, Farideh Jalali-najafabadi, Eftychia Bellou, Pauline Ho, Helena Marzo-Ortega, Philip S Helliwell, Marie Feletar, Anthony W Ryan, David J Kane, Eleanor Korendowych, Michael A Simpson, Jonathan Packham, Ross McManus, Matthew A Brown, Catherine H Smith, Jonathan N Barker, Neil McHugh, Oliver FitzGerald, Richard B Warren, Anne Barton

**Affiliations:** 1 Arthritis Research UK Centre for Genetics and Genomics, Centre for Musculoskeletal Research, Manchester Academic Health Science Centre, University of Manchester, Manchester, UK; 2 Division of Genetics and Molecular Medicine, King’s College London, Guy’s Hospital, London, UK; 3 NIHR Manchester Musculoskeletal Biomedical Research Unit, Manchester Academic Health Science Centre, Manchester, UK; 4 NIHR Leeds Musculoskeletal 12 Biomedical Research Unit, Leeds Teaching Hospitals Trust and Leeds Institute of Rheumatic and Musculoskeletal Disease, University of Leeds, Leeds, UK; 5 Department of Rheumatology, Emeritus Research, Melbourne, Victoria, Australia; 6 Department of Clinical Medicine, Trinity Translational Medicine Institute, Trinity College Dublin, Dublin, Ireland; 7 Adelaide and Meath Hospital and Trinity College Dublin, Dublin, Ireland; 8 Royal National Hospital for Rheumatic Diseases and Department Pharmacy and Pharmacology, University of Bath, Bath, UK; 9 Department of Rheumatology, St Vincent’s University Hospital, UCD School of Medicine and Medical Sciences and Conway Institute of Biomolecular and Biomedical Research, University College Dublin, Dublin, Ireland; 10 Haywood Academic Rheumatology Centre, Institute of Applied Clinical Science, Keele University, Stoke on Trent, UK; 11 Institute of Health and Biomedical Innovation, Queensland University of Technology, Brisbane, Australia; 12 St John’s Institute of Dermatology, Guys and St Thomas’ Foundation Trust, London, UK; 13 St John’s Institute of Dermatology, Division of Genetics and Molecular Medicine, Faculty of Life Sciences and Medicine, King’s College London, London, UK; 14 Dermatology Centre, Salford Royal NHS Foundation Trust, University of Manchester, Manchester, UK

**Keywords:** psoriatic arthritis, psoriasis, MHC, genetic susceptibility, selection bias

## Abstract

**Objectives:**

Psoriatic arthritis (PsA) is a chronic inflammatory arthritis, with a strong heritable component, affecting patients with psoriasis. Here we attempt to identify genetic variants within the major histocompatibility complex (MHC) that differentiate patients with PsA from patients with cutaneous psoriasis alone (PsC).

**Methods:**

2808 patients with PsC, 1945 patients with PsA and 8920 population controls were genotyped. We imputed SNPs, amino acids and classical HLA alleles across the MHC and tested for association with PsA compared to population controls and the PsC patient group. In addition we investigated the impact of the age of disease onset on associations.

**Results:**

HLA-C*06:02 was protective of PsA compared to PsC (p=9.57×10^−66^, OR 0.37). The HLA-C*06:02 risk allele was associated with a younger age of psoriasis onset in all patients (p=1.01×10^−59^). After controlling for the age of psoriasis onset no association of PsA to HLA-C*06:02 (p=0.07) was observed; instead, the most significant association was to amino acid at position 97 of HLA-B (p=1.54×10^−9^) where the presence of asparagine or serine residue increased PsA risk. Asparagine at position 97 of HLA-B defines the HLA-B*27 alleles.

**Conclusions:**

By controlling for the age of psoriasis onset, we show, for the first time, that HLA-C*06:02 is not associated with PsA and that amino acid position 97 of HLA-B differentiates PsA from PsC. This amino acid also represents the largest genetic effect for ankylosing spondylitis, thereby refining the genetic overlap of these two spondyloarthropathies. Correcting for bias has important implications for cross-phenotype genetic studies.

## Background

Psoriatic arthritis (PsA) is a chronic inflammatory arthropathy characterised by spondylitis, enthesitis and arthritis. It is associated with the presence of psoriasis, with a prevalence of up to 14% in this patient group.^1^ The presence of PsA has a substantial impact on a patient’s quality of life, which has been shown to be lower than that of patients with psoriasis alone, partly attributable to increased rates of comorbidities such as cardiovascular disease.^2 3^ The identification of patients with psoriasis at high risk of developing PsA has the potential for significant benefit in patient health as it would allow early intervention to reduce disability and result in an improved outcome for the patient.^4^

Both psoriasis and PsA have a substantial genetic component that influences an individual’s susceptibility; indeed there are now 63  confirmed risk loci for psoriasis in populations of European orign.^5^ The identification of risk loci that are specific for the development of PsA in patients with psoriasis has been more challenging, but evidence is now emerging of loci associated at genome-wide significance thresholds with PsA and not PsC, including loci at 5q31, *IL23R*, *PTPN22*, *TNFAIP3* and *HLA-B*.^6–9^ Genes within the major histocompatibility complex (MHC), in particular HLA class I genes, have been consistently shown to contribute to the susceptibility of both PsC and PsA, with independent associations to *HLA-C, HLA-B* and *HLA-A*.^6 9 10^ Of these, the largest effect is observed with the HLA-C*06:02 allele, where carriage is associated with increased risk and lower age of disease onset of psoriasis.^11^ Interestingly a paradoxical association of HLA-C*06:02 has been reported whereby it is a risk factor for PsA compared with controls, but conversely carriage is protective of PsA within psoriasis.^12–14^ Finally, previous studies have consistently identified the HLA-B*27 and HLA-B*39 alleles as associated with PsA but not PsC, while a more recent analysis of the HLA region based on amino acids rather than genetic haplotypes has reported that an amino acid at position 45 of the mature HLA-B protein is associated with PsA in psoriasis.^9^

The MHC is a particularly challenging region of the genome to map due to the presence of multiple independent associations and extensive linkage disequilibrium between genetic variants. Given the complexity of fine-mapping genetic associations in the MHC, in this study, we attempt to independently validate the previously reported association to the amino acid at position 45 of HLA-B. Here we fine-map genetic associations that differentiate PsA from PsC in large sample collections using imputed single nucleotide polymorphisms (SNPs), classical HLA alleles and amino acid residues.

## Methods

### PsA cohort

A total of 2217 patients with PsA were recruited from rheumatology centres in the UK, Ireland and Australia, as previously described.^6^ PsA classification was defined as the presence of both psoriasis and inflammatory arthritis, regardless of rheumatoid factor status, and all had peripheral arthritis. The majority of patients satisfied the CASPAR (ClASsification criteria for Psoriatic ARthritis) classification system,^15^ although some were collected prior to the introduction of this classification system and all patients were diagnosed by a rheumatologist. All patients provided written informed consent (UK PsA National Repository MREC 99/8/84).

### Psoriasis cohort

We had access to data on 1306 psoriasis patient samples obtained through the Biomarkers of Systemic Treatment Outcomes in Psoriasis study (BSTOP). Patients with severe psoriasis who had also consented to the British Association of Dermatologists Biologics Interventions Registry (a UK pharmacovigilance registry, BADBIR.org.uk) were recruited to BSTOP between October 2011 and October 2015 from 60 secondary and tertiary care outpatient dermatology departments throughout the UK, including centres in London, Manchester, Nottingham and Liverpool. All patients provided written informed consent (BSTOP ethics reference 11/H0802/7). In addition we had access to data on 2622 patients with psoriasis from the Wellcome Trust Case Control Consortium 2 (WTCCC2) study.^16^ Samples from each of these collections were only included in the analysis if they had no previous diagnosis of PsA; we refer to this sample group as cutaneous-only psoriasis (PsC). Classification of PsC in the BSTOP cohort is based on information collected at multiple follow-up consultancies, one every 6 months in the first 3 years and then annually, where an active enquiry of rheumatologist-diagnosed PsA is made. Individuals from the WTCCC2 cohort were excluded based on a known diagnosis of PsA using information provided by sample contributors.

### Population control cohort

A total of 9006 population controls were obtained through the 1958 British birth cohort and the UK Blood Service control group. In addition control data were available from 478 individuals from Ireland.

### Genotyping and quality control

PsA and control population samples were genotyped using the Illumina Immunochip array as previously described, and details are provided in the online supplementary text.^6^ Psoriasis samples were genotyped using the Illumina HumanOmniExpressExome-8v1-2_A array performed at King’s College London. Automated genotype reclustering was performed followed by extensive manual review of genotype clusters based on GenTrain score, cluster separation, allele frequency and call rate. Data for the additional psoriasis samples from the WTCCC2 psoriasis GWAS were generated using the Illumina Human660-Quad genotyping array as previously described.^16^

10.1136/annrheumdis-2017-211414.supp1 Supplementary file 1

### Statistical quality control

Statistical quality control (QC) was performed conforming to established standards in each data set independently. The Immunochip data set (PsA and control samples) was filtered as previously described and details are provided in the online supplementary text.^6^ Statistical QC of the BSTOP data set consisted of the exclusion of samples with a call rate <0.99 and with discrepant sex based on inferred and labelled sex, exclusion of duplicate and related samples using identity-by-state analysis on a set of 75 784 linkage disequilibrium (LD)-pruned SNPs with minor allele freqeuncy (MAF) >0.1 in KING (V.1.4), and exclusion of outliers based on ancestry via principal component analysis (PCA) on the LD-pruned SNPs (also using KING). SNPs were excluded with a call rate <0.99 or Hardy-Weinberg deviation of p<7.5×10^−8^. Both data sets were aligned to the forward strand of the haplotype reference consortium (HRC) reference panel (HRC.r1-1.GRCh37) using the HRC checking tool (http://www.well.ox.ac.uk/~wrayner/tools/). QC of the WTCCC2 data set has been described previously^16^; in addition to this we excluded known PsA samples from the data set, leaving a total of 1784 PsC samples. The data sets were merged and intersecting SNPs were retained. Identity by descent (IBD) was performed on the combined genotype data to identify any overlapping samples.

### Imputation of MHC markers

Imputation of HLA alleles, amino acids and SNPs within the HLA region (chr6:29–34, hg19) was performed with the SNP2HLA software package (V.1.0.3) using the T1DGC reference panel.^17^ Analysis was performed using the imputed dosage on all variants with an information score ≥0.9 and an MAF ≥0.1.

### Statistical analysis

Analysis of all markers was performed using logistic regression assuming an additive effect based on the carriage of alleles. Population structure was controlled for by including the top two principal components as covariates calculated using an intersection of non-HLA SNPs in the combined data set (online supplementary figure S1). For multiallelic sites, such as amino acids, we identified the most common residue or allele in the control population to be selected as the reference and excluded from the model. The p value for each marker was derived from an omnibus test performed with a log-likelihood ratio test of the null and fitted models. Forward stepwise logistic regression was used to identify independent effects where the top marker, ranked by the log-likelihood p value, was included as a covariate by addition to the null model. This was repeated until no further marker reached a predefined significance threshold based on the Bonferroni-corrected type I error rate for the number of markers in the data set. Interactions between the HLA-B*27 allele and non-HLA SNPs were tested in the PsA and control Immunochip data set by fitting an interaction term in the logistic model with HLA-B*27 fitted as a dominant term and the SNP as an additive term.^18^ We tested interactions with rs30187 (*ERAP1*), a previously reported interaction in ankylosing spondylitis (AS), and also to rs12044149 (*IL23R*), rs715285 (5q31), rs2476601 (*PTPN22*) and rs9321623 (*TNFAIP3*), which have previously been reported as differentiating PsA from PsC.^6 8 18 19^ Association of genetic markers with age of psoriasis onset, as a continuous variable, was tested using linear regression, and a difference in the median age of onset between groups was tested using a Wilcoxon test.

## Results

After QC the study data set comprised 1945 PsA cases, 2808 PsC cases and 8920 control samples for 6833 SNPs, 334 amino acids, 71 classical HLA alleles at two-digit resolution and 87 classical HLA alleles at four-digit resolution. A Bonferroni-corrected threshold for p values of 6.8×10^–6^ based on a total of 7325 markers was used to determine significant associations.

### The paradoxical association of HLA-C*06:02 and PsA

**Figure 1:**
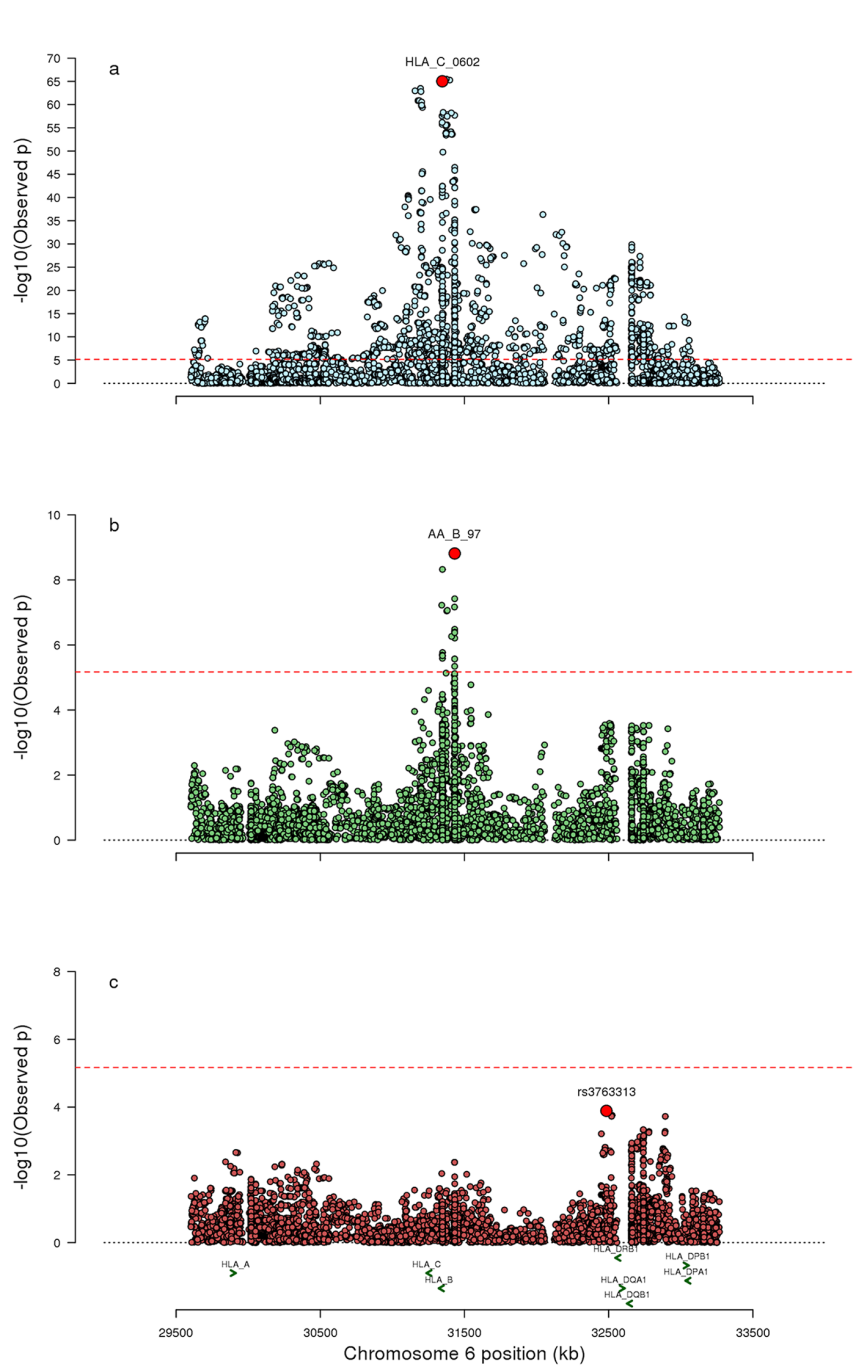
Association results for (A) PsA compared with PsC, (B) PsA compared with PsC controlling for age of psoriasis onset and (C) PsA compared with PsC controlling for age of psoriasis onset and association at amino acid position 97 at HLA-B. Red horizontal line indicates significance threshold; y-axis is –log_10_ of the omnibus test p value, and the x-axis indicates chromosomal base position and gene locations. PsA, psoriatic arthritis.

First, we compared the imputed dosages for all MHC markers for each of the disease groups, PsC and PsA, with the population control group. As expected we replicated the three previously reported independent associations to the class I genes *HLA-C* (HLA-C*06:02), *HLA-B* (amino acid position 67) and *HLA-A* (HLA-A*02:01 or the highly correlated amino acid at position 95, *r*^*2* ^valine=0.99) for each of the diseases (online supplementary figures S2 and S3). We then directly compared PsA with PsC (PsC labelled as the reference group) and observed the most significant association was to the HLA-C*06:02 allele (p=9.57×10^−66^), where the presence of the allele was protective of PsA compared with PsC (OR 0.37, 95% CI 0.33 to 0.41) (figure 1A); the result was in contrast to the previous comparison against controls where the allele was a risk factor for PsA (p=7.44×10^−48^, OR 2.13, 95% CI 1.92 to 2.35). The association remained significant after conditioning on the previously reported HLA-B amino acid position 45 (p=6.88×10^−27^).

### HLA-C*06:02 is associated with the age of onset of psoriasis

Given the previously reported association of HLA-C*06:02 with age of psoriasis onset, we investigated the potential for confounding of the statistical analysis due to selection bias in a data set of 2050 case-only samples with relevant phenotype data (PsA=981, PsC=1069). We found significant association of HLA-C*06:02 allele dose with a younger age of psoriasis onset in all samples (p=1.01×10^−59^; online supplementary figure S4a) and that carriage of the risk allele resulted in a difference in the median age of psoriasis onset of approximately 14 years (online supplementary figure S4b). We found a significant difference (p=1.85×10^−71^) in the median age of psoriasis onset between the PsC (19 years, IQR 15 years) and the PsA groups (34 years, IQR 27 years) (online supplementary figure S5). This result illustrates the potential for confounding when investigating features known to be associated with age of onset as is the case for HLA-C*06:02.

### Psoriasis age of onset confounds HLA analyses

All association analyses comparing PsA with PsC were repeated while conditioning on age of psoriasis onset as a covariate (figure 1B). Within this subgroup of samples, HLA-C*06:02 is significantly associated with a protective effect on PsA (p=4.17×10^−15^, OR 0.52, 95% CI 0.44 to 0.61); however, when conditioning on age of psoriasis onset, there was no evidence of association between HLA-C*06:02 and PsA (p=0.07), suggesting the previously observed protective effect was the result of confounding due to the different age of psoriasis onset in the disease subgroup strata.

### Amino acid position 97 of HLA-B differentiates PsA from PsC

**Figure 2:**
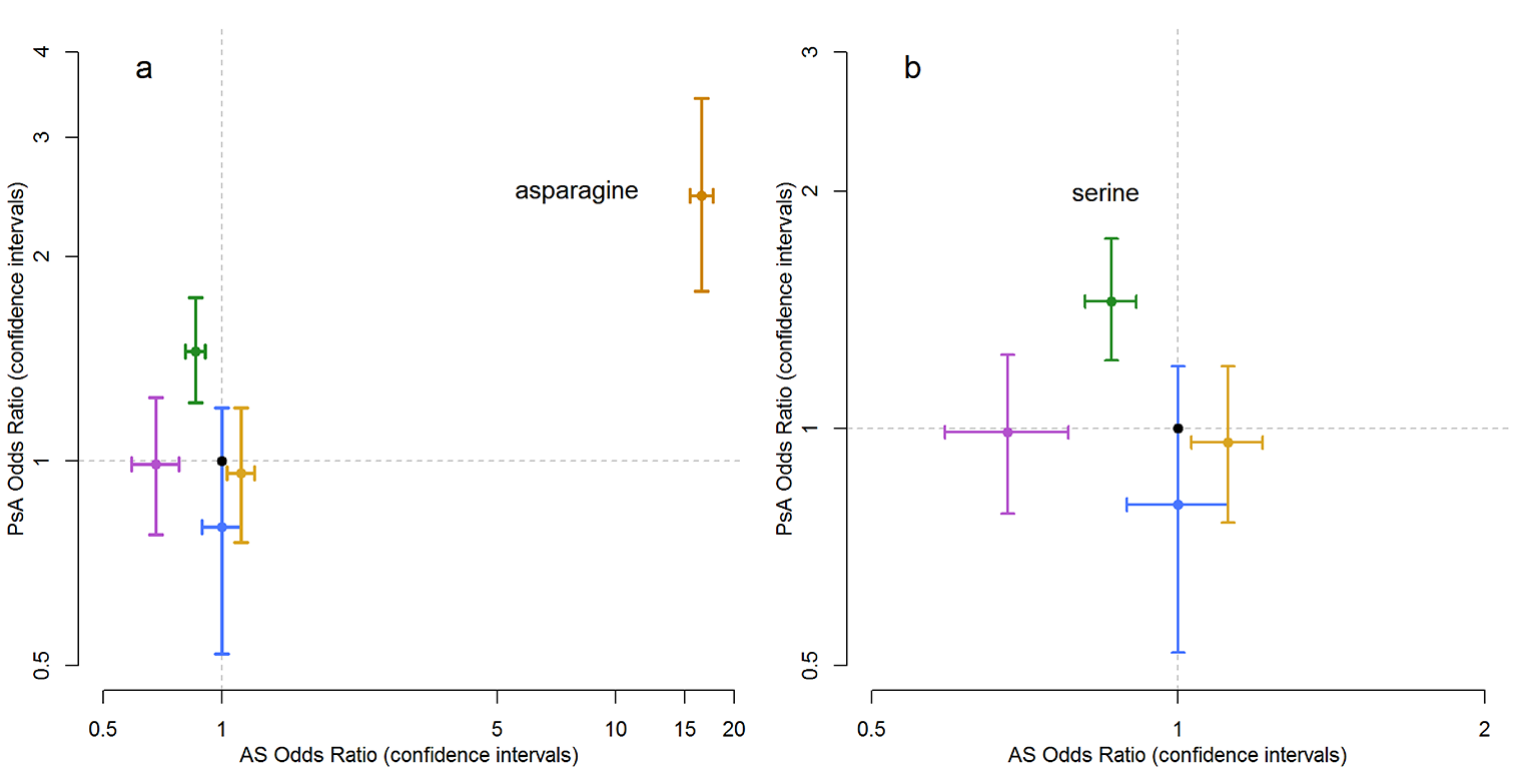
Comparison of effect estimates for residues at amino acid position 97 of HLA-B for PsA and AS showing (A) the asparagine residue is a risk factor for both diseases and (B) the differential effects at the serine residue, which is a risk factor for PsA but protective for AS. AS, ankylosing spondylitis; PsA, psoriatic arthritis.

The most significant association with PsA compared with PsC after correcting for age of psoriasis onset was to an amino acid at position 97 of HLA-B (p=1.54×10^−9^), where the presence of an asparagine (OR 2.46, 95% CI 1.78 to 3.42) or serine (OR 1.45, 95% CI 1.22 to 1.74) residue increased the risk of PsA (table 1). An asparagine residue at position 97 of HLA-B is predominantly found on HLA-B*27 alleles, and HLA-B*27:05 is the most associated HLA allele after correcting for age of psoriasis onset (p=3.53×10^−7^, OR 2.34, 95% CI 1.69 to 3.25); in addition, a serine residue is found on multiple HLA alleles including HLA-B*07 (p=1.9×10^−3^) and HLA-B*08 (p=0.05). However neither of these two alleles were independently associated with PsA when conditioning on amino acid position 97 (p>0.05), while amino acid 97 remained associated independently of either of these two HLA alleles and was independently associated when adjusting for HLA-B*27, indicating that amino acid 97 is the primary driver of the associations observed with these HLA-B alleles. This amino acid is an important risk factor for AS; comparison of effect estimates shows that an asparagine residue increases risk for both diseases, although with a substantially larger effect estimate in AS (OR 16.51, 95% CI 15.43 to 17.69) than PsA (OR 2.46, 95% CI 1.78 to 3.42) (figure 2).^20^ In contrast, the presence of a serine residue is associated with risk of PsA (OR 1.45, 95% CI 1.22 to 1.74) while reported to have a protective effect for AS (OR 0.86, 95% CI 0.81 to 0.91).

**Table 1:** Summary statistics for residues of amino acid at position 97 of HLA-B and association with psoriatic arthritis compared with cutaneous psoriasis alone

Residue	Amino acid	Frequency	p Value	OR	95% CI
R	Arginine	0.4619	Ref	Ref	Ref
S	Serine	0.2785	3.58E-05	1.45	1.22:1.74
T	Threonine	0.1003	0.716	0.959	0.76:1.20
V	Valine	0.0751	0.913	0.988	0.78:1.24
N	Asparagine	0.0474	5.76E-08	2.46	1.78:3.42
W	Tryptophan	0.0384	0.283	0.795	0.52:1.20

p Value and ORs are determined with multivariate logistic regression.

We found significant association to the previously reported amino acid at position 45 of HLA-B (p=3.5×10^−4^; online supplementary table S1); however, this was not significant after adjusting for amino acid position 97 (p=0.16). No further associations exceeded the significance threshold when conditioning on amino acid 97 (figure 1C). We found no evidence to support the previously reported interaction between HLA-B*27 and *ERAP1* observed in AS or with any of the other previously reported PsA differentiating loci (p value >0.05).

## Discussion

Through detailed analysis of the MHC region using data from patients with PsA, PsC and population controls, we show that, first, previous reports of a protective effect of HLA-C*06:02 with PsA are due to confounding by differences in the age of onset of psoriasis due to the strong association of HLA-C*06:02 with a younger age of psoriasis onset; second, HLA-C*06:02 and HLA-A*02:01 are primarily associated with psoriasis and confer no additional risk of PsA; and, third, that when age of psoriasis onset is accounted for, the primary association conferring additional risk for PsA in patients with psoriasis is to the presence of asparagine (HLA-B*27) or serine (HLA-B*07 and HLA-B*08) residues at amino acid position 97 of HLA-B.

Understanding the genetic factors that differentiate PsA from PsC is important both for screening patients at risk for psoriasis and for understanding the disease mechanisms involved. In terms of screening, given that psoriasis often predates PsA, factors that identify a group of patients with psoriasis at higher risk of developing PsA could potentially allow the introduction of preventative strategies in the future. Indeed, the application of genetic risk scores in high-risk groups where disease prevalence is much higher than the general population has been shown to greatly increase the diagnostic benefit of genetic risk factors.^21^ At a practical level, however, while genotyping costs have improved, analysis and interpretation of HLA data from genotyping arrays remain time-consuming and challenging, and it is still not clear how much more information is provided over and above classical HLA typing methods. Thus, if HLA screening were shown to be useful in prospective studies of patients with psoriasis, HLA typing for HLA-B*27 may remain the preferred option.

Okada *et al* report residues at the amino acid position 45 of HLA-B as the key risk factor for PsA in psoriasis; however, the current study does not support this after correcting for the age of psoriasis onset (p value=3.5×10^−4^). PsA is a clinically heterogeneous disease and one possible explanation of this discrepancy is differing proportions of clinical subgroups between the studies. For example HLA-B*27 has been reported to be associated with axial disease within a well-phenotyped PsA patient cohort^22^; therefore, the current study may be enriched for axial disease. This highlights the need for accurate clinical phenotyping of PsA cases, and one of the major limitations of the current study was that this could not be investigated further due to the lack of information about the presence of axial disease in all of our patients with PsA.

Amino acid position 97 of HLA-B represents the largest genetic effect reported in AS^20^; our analysis has confirmed the genetic overlap of PsA with AS. It could be argued that PsA may simply be an overlap of AS and psoriasis, but clinical, radiographic and genetic differences have been observed. For example, methotrexate is more effective in PsA than AS; classical pencil-in-cup deformities, osteolysis and juxta-articular new bone formation in hands and feet are more common in PsA, and genetic variants have been identified that are associated with one disease but not the other; for example, PsA variants at the *IL23R* are distinct from those reported for AS.^6^ Amino acid residues at position 97 are the most important risk factor for both diseases, and our results highlight both overlapping and differential associations. The asparagine residue is associated with increased risk in both diseases; however, the serine residue has a differential association representing increased risk for PsA while being protective of AS. The position is located within the peptide binding groove of the HLA-B molecule and highlights the importance of antigen presentation in disease aetiology. We were unable to replicate the interaction of HLA-B*27 and *ERAP*1 observed in AS.^18^ This may be due to insufficient power of the current study to detect an interaction due to the lower effect sizes in PsA or could indicate differing disease mechanisms.

Our study highlights the importance of accounting for confounding in genetic studies, particularly when associated loci are correlated with timing of disease onset. We believe the confounding observed in this study is due to ascertainment bias where cases of type I psoriasis, age of onset <40 years, are preferentially included in genetic studies and such selection does not occur in PsA collections. The issue of selection bias is increasingly being recognised in the statistical methodology literature.^23 24^ In particular, index event bias describes how conditioning on an outcome, for example psoriasis, can induce correlation between risk factors leading to spurious associations.

In conclusion, we show that HLA-C*06:02 is primarily associated with psoriasis with no effect, either risk or protective, on PsA, while HLA-B amino acid 97, the same variant that represents the major AS risk factor, is the most important risk factor for PsA.
