# Supplementary material for: Cross-phenotype association mapping of the MHC identifies genetic variants that differentiate psoriatic arthritis from psoriasis
Source: Ann Rheum Dis. 2017 Aug 18;76(10):1774–9. doi: 10.1136/annrheumdis-2017-211414 (PMC5629941; doi:10.1136/annrheumdis-2017-211414)
Supplement: Supplementary file 1 [file annrheumdis-2017-211414supp001.docx]

**Immunochip dataset**

*Genotyping and quality control*

Genotyping was performed at five centres; the Wellcome Trust Sanger Institute, the Centre for Public Health Genomics at The University of Virginia, Arthritis Research UK Centre for Genetics and Genomics at The University of Manchester, the University of Queensland Diamantina Institute, and the Trinity Translational Medicine at The University of Dublin. Genotype clustering and calling of all Immunochip data was performed at the Arthritis Research UK Centre for Genetics and Genomics using the GenomeStudio Data Analysis software platform (Genotyping Module v1.8.4). Automated genotype reclustering was performed following exclusion of poor quality samples (call rate < 0.9) and manual inspection of clusters was performed based on cluster separation (<0.4), signal intensity (<1.0), call rate (<0.98) and allele frequency.

*Statistical quality control*

Samples were excluded with a call rate < 0.98 or if they were identified as being an outlier based on autosomal heterozygosity (3 standard deviations from the mean autosomal heterozygosity of samples passing the call rate threshold). Duplicate and related individuals were identified using identity-by-descent (IBD), performed in PLINK (v1.07) using a set of 18,664 LD pruned (*r*^2^) SNPs with a minor allele frequency (MAF) > 0.05. Principal component analysis (PCA), using EIGENSOFT (v4.2) and the LD pruned SNP set, was performed to infer ancestry and outliers were manually removed. SNP filtering was performed on the remaining high quality samples; this included a call rate threshold of ≥0.98 in cases and controls, exclusion of SNPs with significant deviation from Hardy-Weinberg equilibrium (HWE) p-value < 5x10^-7^ in controls, and a minor allele frequency (MAF) < 0.01.

**
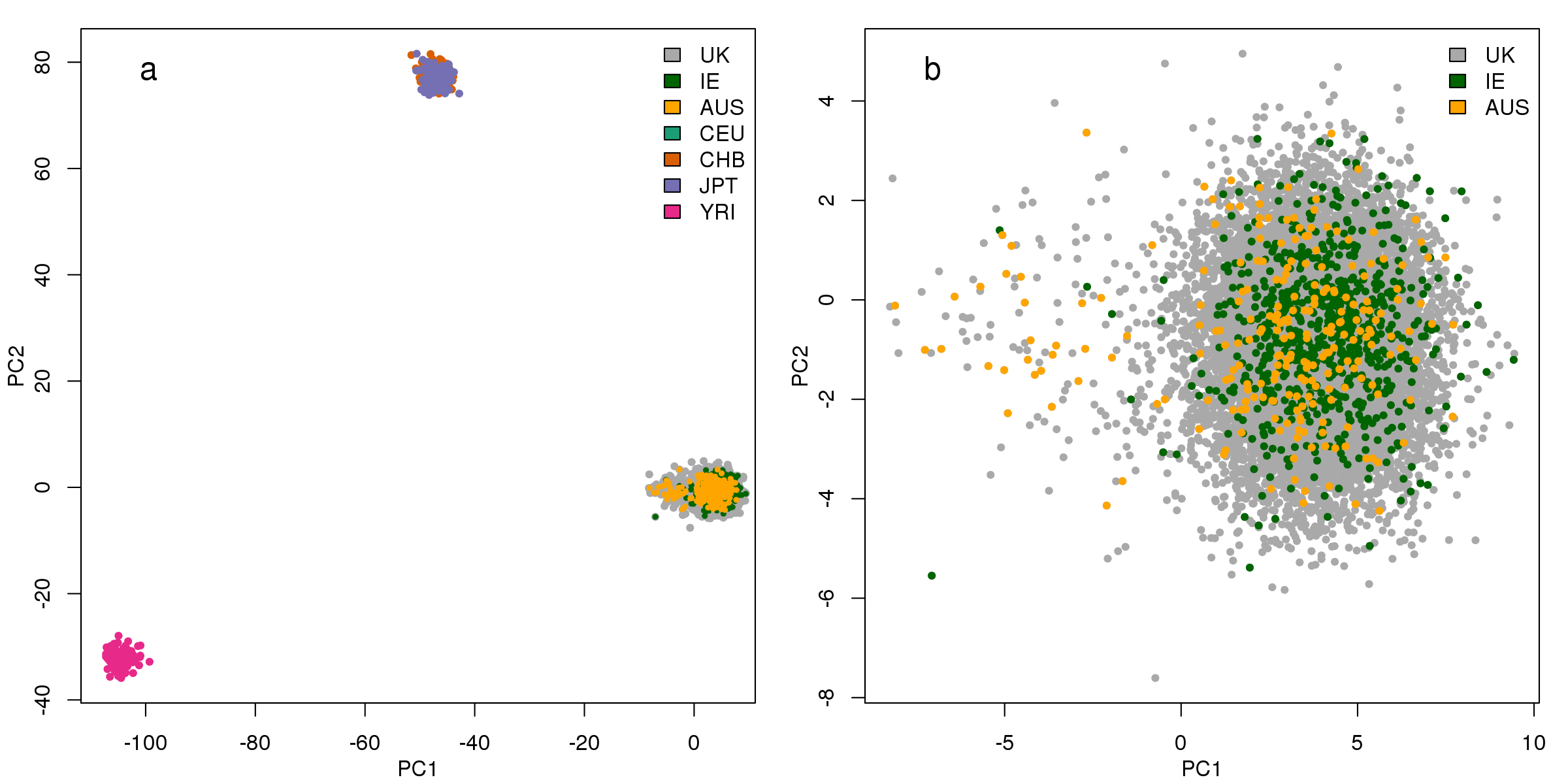
**

**Supplementary Figure S1:** Principal components calculated using flashpca based on X single nucleotide polymorphisms for a) study samples plus CEU, CHD, JPT and YRI HapMap3 data and b) study sample only. PC; principal component, UK; United Kingdom, IE; Republic of Ireland, AUS; Australia, CEU; Utah residents with Northern and Western European ancestry from the CEPH collection, CHB; Han Chinese in Beijing, China, JPT; Japanese in Tokyo, Japan, YRI; Yoruba in Ibadan, Nigeria.

**
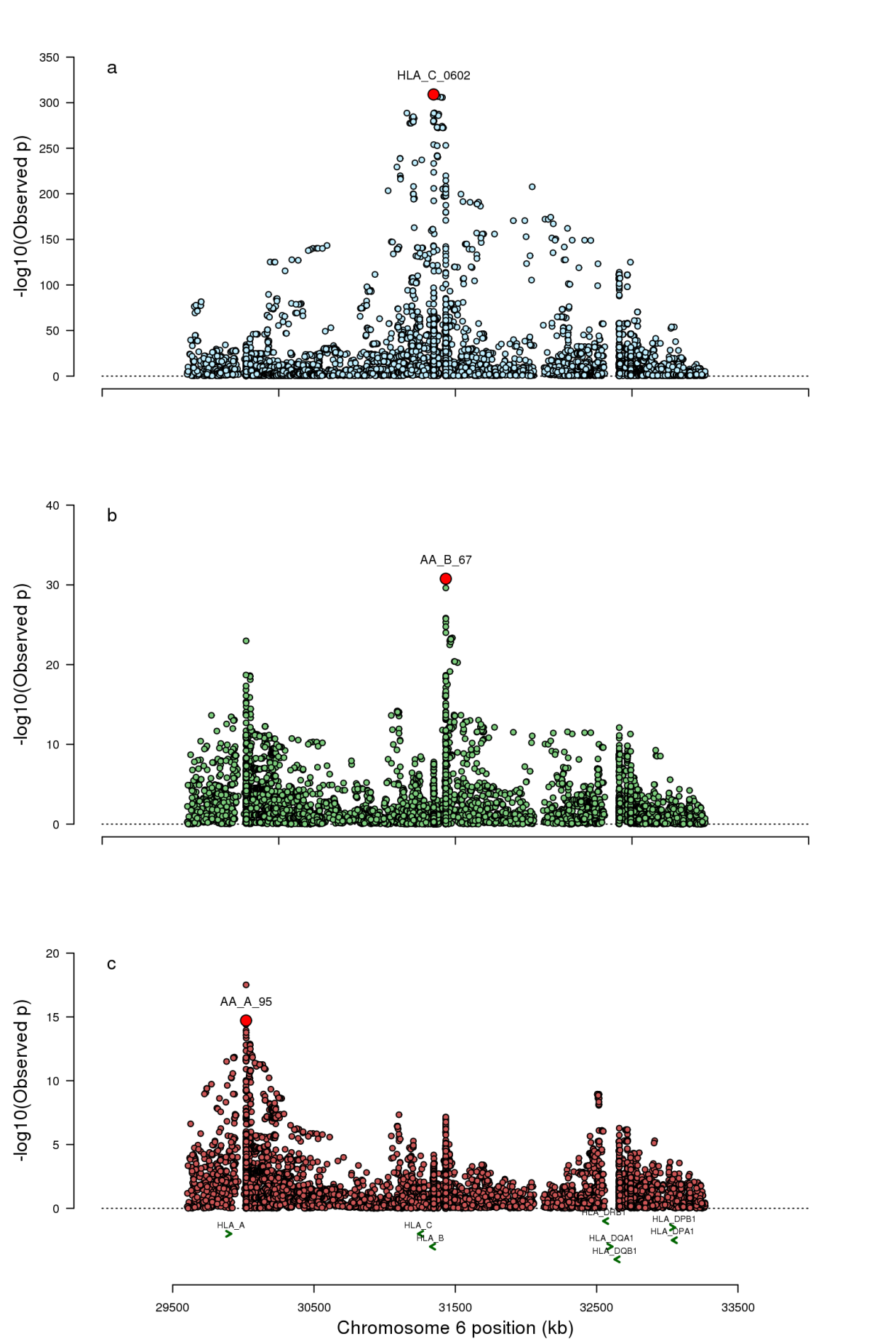
**

**Supplementary Figure S2:** Association of markers across the MHC for psoriasis compared to controls. Stepwise forward logistic regression confirms three independent associations at *HLA-C* (a), *HLA-B* (b) and *HLA-A* (c), y-axis is –log_10_ of the omnibus test p-value and the x-axis indicates chromosomal base position and gene locations.


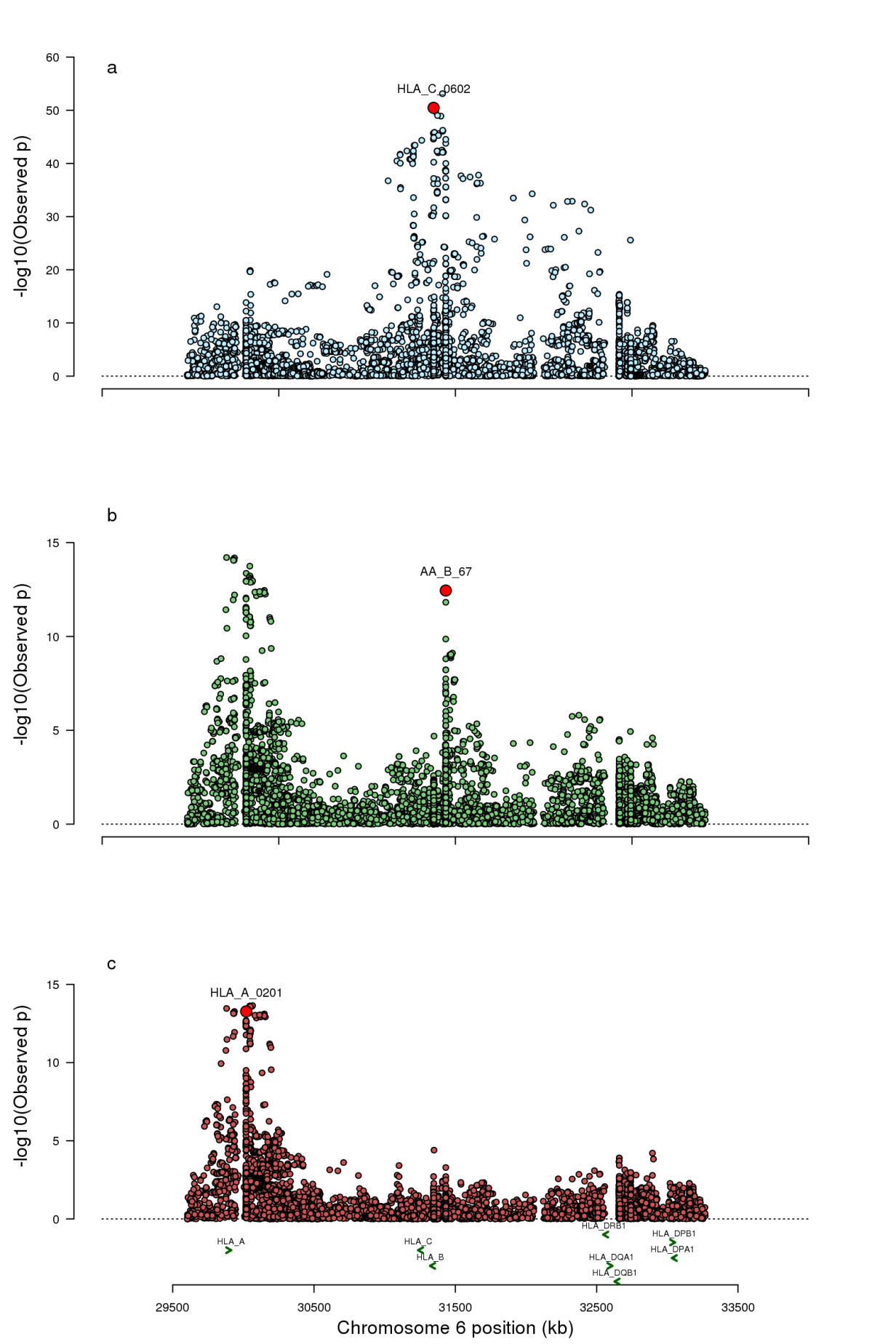


**Supplementary Figure S3:** Association of markers across the MHC for PsA compared to controls. Stepwise forward logistic regression confirms three independent associations at *HLA-C* (a), *HLA-B* (b) and *HLA-A* (c), y-axis is –log_10_ of the omnibus test p-value and the x-axis indicates chromosomal base position and gene locations.


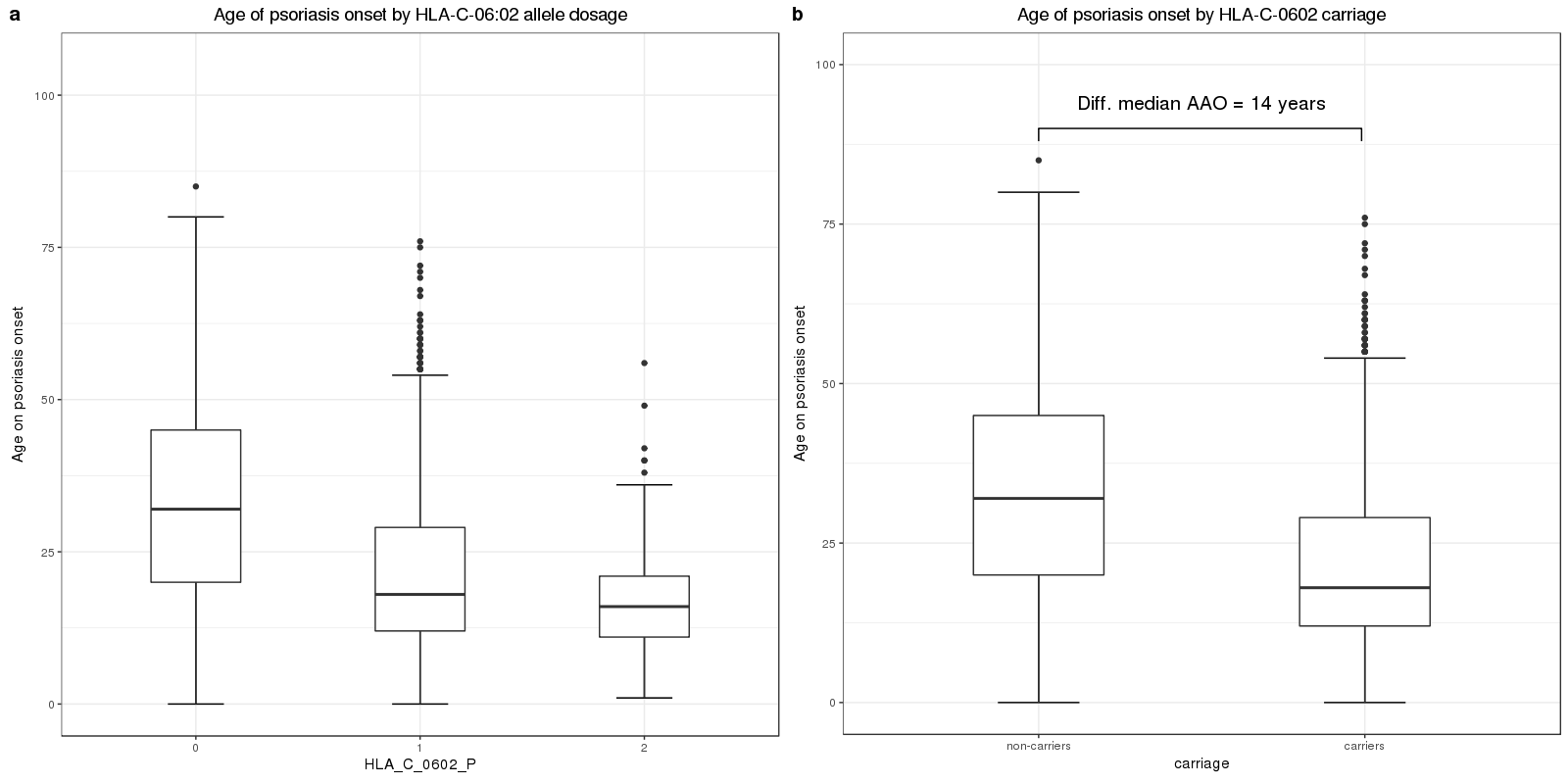


**Supplementary Figure S4:** Boxplots illustrating age of psoriasis onset by a) *HLA-C*06:02* allele dosage where the number of risk alleles carried (x-axis) is associated with a younger age of psoriasis onset (p-value 1.01x10^-59^) tested by linear regression and, b) by *HLA-C*06:02* carriage showing difference in the median age of psoriasis onset of approximately 14 years with carriage of the risk allele.


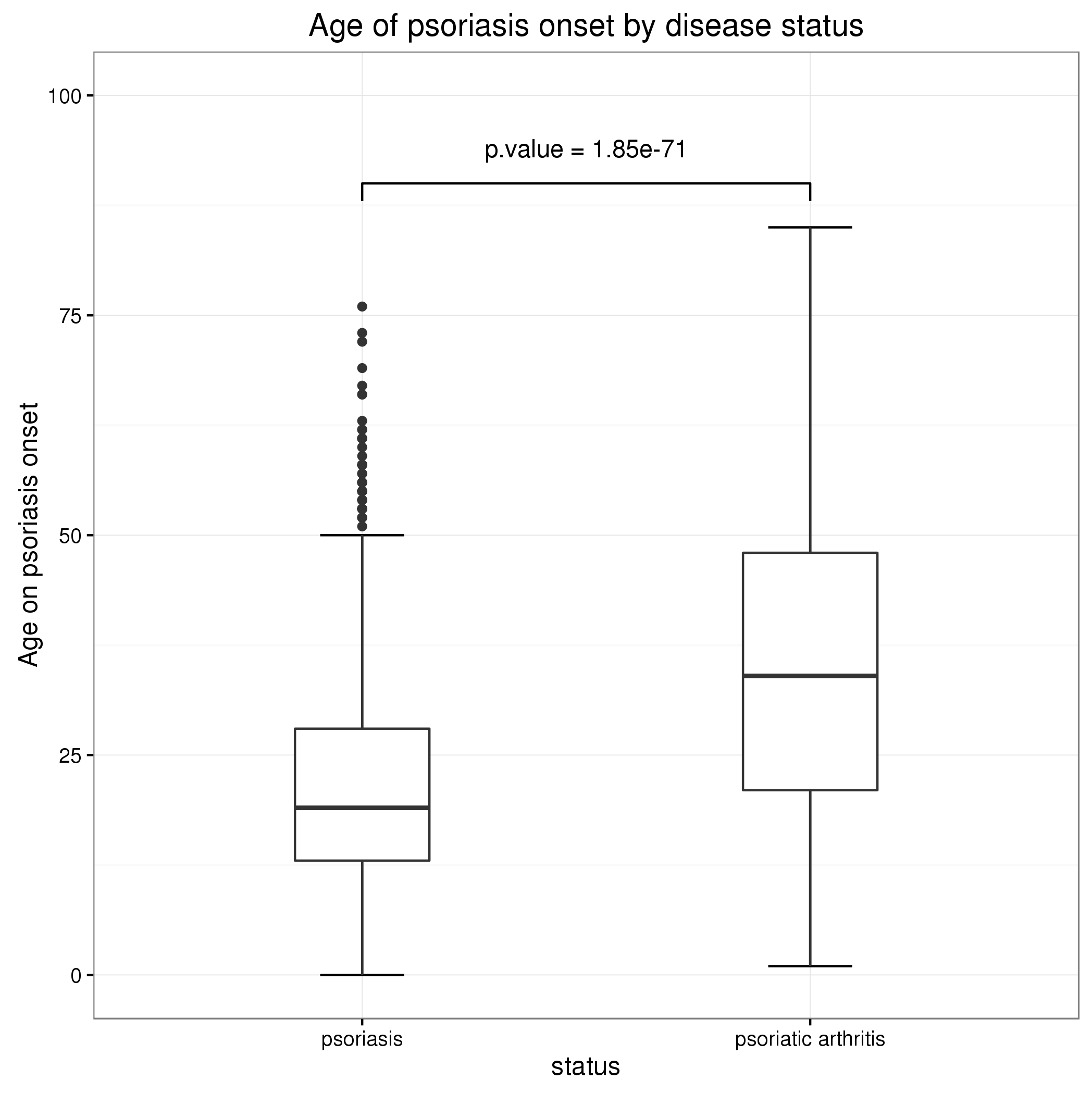


**Supplementary Figure S5:** Boxplot illustrating differences in the age of psoriasis onset between the PsA and cutaneous only psoriasis subgroups. The cutaneous only psoriasis study group had a significantly lower age of psoriasis onset.

| Residues | Amino acids | Frequency | P-value | Odds ratio | 95% confidence interval |
| --- | --- | --- | --- | --- | --- |
| E | Glutamic acid | 0.42 | 1.20E-04 | 1.32 | 1.15:1.52 |
| T,K,M | Threonine, Lysine, Methionine | 0.58 | Ref | Ref | Ref |

**Supplementary Table S1:** Summary statistics for residues of the amino acid at position 45 of HLA-B for association with PsA compared to cutaneous psoriasis alone. P-value and odds ratio are determined with multivariate logistic regression for the glutamic acid residue compared to a combined reference group of threonine, lysine and methionine.
